# Psychological Barriers to the Use of Opioid Analgesics for Treating Pain in Patients With Advanced Recurrent Cancer: A Multicenter Cohort Study

**DOI:** 10.1089/pmr.2023.0068

**Published:** 2024-01-19

**Authors:** Takehiko Tsuno, Takashi Kawaguchi, Ryota Yanaizumi, Junichi Kondo, Keiko Kojima, Takashi Igarashi, Masaki Inoue, Tomofumi Miura, Akime Miyasato, Kanako Azuma, Hiroshi Hamada, Tomoya Saeki, Hironori Mawatari, Hiroyuki Ogura, Akira Kotani, Takuhiro Yamaguchi, Hideki Hakamata

**Affiliations:** ^1^ Department of Pharmacy, Yokohama City University Medical Center, Yokohama, Japan.; ^2^ Department of Analytical Chemistry, Tokyo University of Pharmacy and Life Sciences, Tokyo, Japan.; ^3^ Department of Practical Pharmacy, Tokyo University of Pharmacy and Life Sciences, Tokyo, Japan.; ^4^ Department of Anesthesiology, Yokohama City University Medical Center, Yokohama, Japan.; ^5^ Department of Palliative Medicine, Yokohama City University Medical Center, Yokohama, Japan.; ^6^ Department of Pharmacy, National Cancer Center Hospital East, Kashiwa, Japan.; ^7^ Department of Palliative Medicine, National Cancer Center Hospital East, Kashiwa, Japan.; ^8^ Department of Pharmacy, Tokyo Medical University Hospital, Tokyo, Japan.; ^9^ Department of Palliative Medicine, Tokyo Medical University Hospital, Tokyo, Japan.; ^10^ Department of Pharmacy and Yokohama Minami Kyousai Hospital, Yokohama, Japan.; ^11^ Department of Palliative and Supportive Care, Yokohama Minami Kyousai Hospital, Yokohama, Japan.; ^12^ Department of Pharmacy, Kameda Medical Center, Chiba, Japan.; ^13^ Division of Biostatistics, Tohoku University Graduate School of Medicine, Sendai, Japan.

**Keywords:** cancer pain, decision regret, psychological barriers, strong opioids

## Abstract

**Background:**

We aimed to gain insight into psychological barriers toward initiation of strong opioid analgesic use in patients with advanced recurrent cancer.

**Methods:**

This study included 46 patients who were prescribed with opioid analgesics for advanced recurrent cancer. The primary outcome was psychological barriers assessed using the Japanese version of the Barriers Questionnaire-II (JBQ-II). The secondary outcomes were psychological changes and pain relief one week after the induction of strong opioid analgesics.

**Results:**

The mean age of participants was 63.6 years. Furthermore, 26.1% had an Eastern Cooperative Oncology Group (ECOG) performance status of ≥3. The mean JBQ-II total score was 1.97 (95% confidence interval: 1.75–2.19). At the initiation of opioid therapy, there was no difference in the total scores between the baseline and one week later. Nevertheless, there was a significant difference in the subscale “disease progression” score (mean 2.97 vs. 2.59, difference in means 0.38, standard error 0.16, *p* = 0.026). Personalized Pain Goal (PPG) was achieved in about half of the participants, and a trend toward a higher score in the subscale “harmful effects” (concern about adverse events) was observed in those who did not achieve PPG.

**Conclusion:**

This study showed that patients with advanced recurrent cancer have psychological barriers to opioid induction. The relationship between the presence of psychological barriers before and after induction of opioid analgesics and the speed of pain improvement was determined. The results may provide fundamental information for prospective intervention studies to develop individualized education programs for patients with psychological barriers to opioids.

Clinical Trial Registration Number UMIN000042443.

## Background

Pain is the most common symptom in patients with cancer. Approximately 80% of patients with advanced cancer experience moderate to severe pain,^1^ and they often hesitate to manage their pain using opioid analgesics.^2^ The Barriers Questionnaire (BQ) quantitatively measures factors related to patients' hesitation in using opioids.^3^ Psychological barriers are lower when analgesics are appropriate for the pain level as compared with inadequate analgesics.^4–6^

In a Japanese questionnaire study, 28% patients with advanced and recurrent cancer believed that opioid analgesic use shortens their lifespan and causes addiction.^7^ Meta-analysis showed that Asians have greater barriers to cancer pain progression, tolerance, and lethality than Westerners.^8^ In addition, using a questionnaire, regulatory authorities of various countries found a high percentage of patients with concerns about dependency in East Asia, which includes Japan.^9^ Weak and strong opioids have different regulatory perspectives and patient perceptions.

In Japan, the indications for strong opioid analgesics are more strictly regulated than in other countries, and their use is limited to pain relief for conditions other than cancer pain,^10^ and a certain number of patients in Japan were considered pain relief with strong opioid analgesics to be “hastening death” or a “last resort.”^11^ Therefore, patient consideration should be given when induction of strong opioid analgesics, even in patients who have been previously exposed to weak opioid analgesics. Patients who had used strong opioid analgesics for short periods showed higher psychological barrier scores than those who had used them for longer periods.^12^

Despite these barriers, acceptance of opioid use for pain relief is expected to improve through the practice of high-quality palliative care, pain relief after administration of narcotic medication, and through improved confidence in drug safety.^13^ However, psychological barriers to the use of strong opioid analgesics at treatment initiation have not been investigated in patients with advanced recurrent cancer. Therefore, it would be useful to investigate the relationship between pain relief and psychological changes immediately after drug administration. These results are expected to provide essential information to the development of an individualized education program for patients with significant psychological barriers.

The purpose of this study was to identify psychological barriers to the induction of strong opioid analgesics in Japanese patients with advanced recurrent cancer. We hypothesized that patients have substantial psychological barriers and are affected by pain relief and psychological changes immediately after drug administration.

## Methods

### Ethical consideration

The protocol was developed according to the STROBE Statement^14^ and SPRIT-PRO.^15^ Institutional review board approval was obtained at all study sites.^16^ Before enrolment, an investigator explained the details of the study to the patients. Informed consent was obtained from all participants. This study was registered with the University Hospital Medical Information Network Clinical Trials Registry (UMIN-CTR) in Japan (Trial registration number UMIN000042443).

### Study design and patients

This is a multicenter longitudinal observational study. The inclusion criterion was distant metastasis or advanced recurrent cancer in patients who received strong opioid analgesics for cancer pain during their first treatment. Patients with the highest pain severity in the last 24 hours with a Numerical Rating Scale (NRS) score of ≥4 were included. We have defined morphine, oxycodone, fentanyl, hydromorphone, and tapentadol as strong opioid analgesics for the purposes of this study.

In addition, strong opioid analgesics were prescribed in any dose. The exclusion criteria were (1) patients who had difficulty operating a “bring your own device” (BYOD) to collect electronic patient-reported outcomes (ePRO) (e.g., those who do not have a smartphone or cannot use a tablet), (2) those with cognitive or psychiatric disorders that would interfere with PRO administration, (3) those whose primary pain mechanism was neuropathic, and (4) other factors that the attending physician deems inappropriate.^16^

Because titration of strong opioid analgesics can be assessed in one week,^17^ the observation period was set at seven days to investigate psychological barriers at the start of treatment and pain relief.

### Measurement tools

#### The Japanese version of the Barriers Questionnaire II

To reflect practical changes in pain management, BQ, which is a measure of psychological barriers, was revised to create the Barriers Questionnaire II (BQ-II).^5^ The Japanese version of the BQ-II (JBQ-II) has been validated earlier.^18^ The JBQ-II comprises the following five subscales: barriers related to psychological effects (distrust of symptomatic treatment, fatalism [fateful resignation], communication [loss of intention], adverse effects [fear of side effects], and disease progression [escape/defense from illness]). Each item was graded on a 6-point Likert scale (0–5). The subscale and total scores (overall barriers) were calculated as the mean of the scores (0–5) for the relevant items, with higher numbers indicating increased barriers.

#### Decision Regret Scale

Regret is a negative emotion experienced when one realizes or imagines that one has made the wrong choice. It is a retrospective unpleasant feeling, and people tend to focus on “what is good” rather than “what is bad.” It has been reported to be associated with negative emotions, such as disappointment, and involve some aspect of self-blame.^19^ Patient regret during a strong opioid treatment process was assessed using the Decision Regret Scale (DRS), which measures regret experienced by the patients regarding decisions made during the treatment.^20^

A Japanese version of the DRS has been previously developed and validated.^21^ It consists of five items: (1) It was the right decision, (2) I regret the choice that was made, (3) I would go for the same choice if I had to do it over again, (4) the choice did me a lot of harm, and (5) the decision was a wise one. Items are scored on a 5-point Likert scale (1–5). Scores were reversed for items (2) and (4); mean scores were obtained and then converted by subtracting 1 and multiplying by 25. The total score ranges from 0 to 100, with higher scores indicating greater regret.

#### Brief Pain Inventory-Short Form

The Brief Pain Inventory (BPI) is a reliable and valid scale for assessing pain intensity and its effect on daily life.^22^ It is a 15-item questionnaire used to evaluate pain. Each item is graded on an 11-point scale, with scores ranging from 0 to 10. The Japanese version of this scale has been validated, and its reliability and usefulness have been established.^23^ To decrease the burden on patients related to the number of questions to be answered, this study used only the “worst pain in the last 24 hours” item from the BPI-Short Form (BPI-SF) based on a report by Atkinson et al.^24^

#### Personalized Pain Goal

From the perspective of personalized medicine for the treatment of cancer pain, it is important to involve the patient in treatment goal setting and provide treatment with the aim of achieving those goals. Personalized Pain Goal (PPG) is a pain relief goal that is self-defined by individual patients and has been used as an outcome measure to determine pain-relief goals in patients with cancer.^25^ The PPG helps patients set a personalized pain-relief goal using the following question: “At what level would you feel comfortable with the pain?”^26^

In our study, patients were asked to use the NRS to indicate their pain treatment goals. PPG attainment was defined as the first time when the worst pain item on the BPI was equal to or below the patient's established PPG with the opioid prescription date (synonymous with the start date) as the baseline (day 0) and up to day 7. The period of PPG attainment was defined as the period from baseline (day 0) to the day PPG was attained. Successful pain treatment (achievement of PPG) was defined as pain being below PPG.

#### Pain Management Index

The Pain Management Index (PMI) is a frequently used and well-validated tool for assessing the adequacy of pain management in patients experiencing pain or in those under treatment with analgesics.^27^ It is calculated by subtracting the patient-rated pain score from the analgesic drug score.^28^ Negative PMI scores indicate inadequate pain management, whereas scores of ≥0 reflect adequate pain management.

#### Patient Global Impression Scale-Severity

Currently, the cutoff values for classifying the presence and magnitude of psychological barriers to opioid analgesics are unknown. This study used the Patient Global Impression Scale-Severity (PGI-S) scale to classify the participants' JBQ-II scores. The PGI-S has not yet been validated for classifying psychological barriers.

Responses to the item “At present, how reluctant are you to use opioids for pain relief?” were scored on the following 7-point scale: 0 (not at all), 1 (not reluctant), 2 (almost not reluctant), 3 (neither), 4 (slightly reluctant) 5 (reluctant), and 6 (extremely reluctant).

### Data collection and timeline

Data regarding psychosocial background, JBQ-II and BPI-SF scores, and PPG were collected from the participants (day 0). Furthermore, ePRO was collected using BYOD. Data regarding demographics, medical history, and PMI were collected by investigators, entered into the web-based EDC system at the study site, and linked to the baseline PRO data. After starting opioid administration, participants were asked to record their BPI-SF (worst pain in the last 24 hours) daily for 7 days (days 1–7).

The pain was assessed using ePRO for appropriate daily pain assessment in both inpatients and outpatients. The JBQ-II, PGI-S, and DRS were administered on the last day (day seven). (Table 1) During the first week of treatment, observation was discontinued if death or worsening of the condition during monitoring made it impossible to continue the study. Pain management and supportive care provided to patients enrolled at each center were not specified.

**Table 1.** Study Procedure and Time Points for Evaluations

Day	Visit 1	Time after initiating opioid therapy							Visit 2
	0 (baseline)	1	2	3	4	5	6	7	8–15
Psychosocial background	●								
The Japanese version of the Barriers Questionnaire II	●							●	
DRS								●	
Brief Pain Inventory-Short Form (strongest pain in the past 24 hours)	●	●	●	●	●	●	●	●	
PPG	●								
PGI-S								●	
CTCAE v5.0-JCOG	●								●
PRO-CTCAE	●							●	

DRS, Decision Regret Scale; PGI-S, Patient Global Impression Scale-Severity; PPG, Personalized Pain Goal; ●, time point for evaluation.

### Outcomes

The primary outcome was the baseline JBQ-II score. The mean JBQ-II score at baseline and its confidence interval (95% CI) were calculated. Second, the relationships between the total JBQ-II score and PPG achievement period, baseline and second visit JBQ-II scores, changes in JBQ-II scores, and PPG achievement rate on day seven were examined. Patients were grouped based on their PGI-S scores, and the difference between the DRS scores and PPG achievement rates was estimated and tested. The relationship between the JBQ-II scores and trends in pain scores was investigated.

### Sample size

Initially, the sample size was set at 200.^16^ However, after the study was initiated, there were difficulties in collecting cases due to the number of eligible patients who were administered strong opioid analgesics decreased by 20% in 2020 as compared with the previous year, the difficulty in obtaining consent due to general conditions at the time of administration, and the difficulty in finding eligible patients with opioid prescriptions outside of palliative care outpatient clinics. In light of this, the number of cases that could be registered at other facilities was reconfirmed. Therefore, the target enrolment number was set at 50 cases.

### Statistical analysis

For all enrolled cases, summary statistics (number of observations, mean, and standard deviation) were calculated for demographic variables, baseline values (patient characteristic data before the start of observation) for numerical variables, and frequencies and proportions for each level for categorical variables. This study did not supplement missing data. Variables with missing values were left as is, and the analysis was based only on the observations obtained (observed case analysis). Statistical significance was set at *p* < 0.05. We decided not to adjust for multiple comparisons due to the exploratory nature of this study. All analyses were performed using JMP Pro 15 (SAS Institute, Inc., Cary, NC).

## Results

### Participant flow and characteristics

**FIG. 1.**
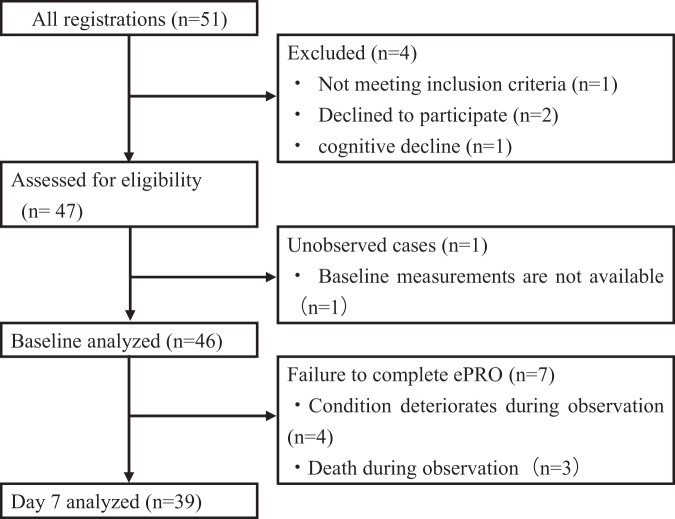
Participant flow diagram, according to STROBE guidelines. ePRO, electronic patient-reported outcomes.

Over the study period (October 2020 and December 2021), 51 participants scheduled for induction of strong opioid analgesics were enrolled. A total of 47 participants met the inclusion criteria, and 4 were excluded. Among the 47 participants who met the eligibility criteria, 1 could not be evaluated at baseline, with a final count of 46 participants. These participants were assessed at baseline, and 39 (84.8%) completed the survey (responded up to day seven) (Fig. 1).

The mean (standard deviation) age was 63.6 (11.1) years, 13 participants (28.3%) were outpatients, 18 participants (39.1%) had previous received weak opioid analgesics, and 35 participants (76.1%) had received anticancer treatment (radiotherapy, chemotherapy, and chemoradiotherapy). The study enrolled 12 participants (26.1%) with an Eastern Cooperative Oncology Group (ECOG) performance status (PS) ≥3 (Table 2).

**Table 2.** Baseline Characteristics (*n* = 46)

	***n*** (%)
Age: mean, SD	63.6, 11.1
Sex	
Male	34 (73.9)
Female	12 (26.1)
Primary tumor site	
Gastrointestinal^a^	23 (50.0)
Lung	6 (13.0)
Prostate	4 (8.7)
Head and neck	4 (8.7)
Gynecology	3 (6.5)
Other	6 (13.0)
Metastasis	
Yes	36 (78.3)
No	10 (21.7)
Under anticancer treatment^b^	
Yes	35 (76.1)
No	11 (23.9)
ECOG performance status	
0–2	34 (73.9)
3–4	12 (26.1)
Environment	
Inpatient	33 (71.7)
Outpatient	13 (28.3)
Pain Management Index	
−3	1 (2.2)
−2	15 (32.6)
−1	18 (39.1)
0	12 (26.1)
Education (*n* = 44)	
High	7 (15.9)
Middle	31 (70.4)
Low	5 (11.3)
Other	1 (2.3)
Employment status (n = 44)	
Full time	15 (34.1)
Part time	4 (9.1)
Other	25 (56.8)
Marital status (*n* = 44)	
Single	9 (20.5)
Married	26 (59.1)
Divorced	3 (6.8)
Bereaved	6 (13.6)

^a^ Colon, rectum, esophagus, stomach, and pancreas.

^b^ Radiotherapy, chemotherapy, molecularly targeted therapy, immunotherapy, and hormone therapy.

ECOG, Eastern Cooperative Oncology Group; SD, standard deviation.

### Attitudinal barriers to opioid analgesics (total JBQ-II scores)

The mean JBQ-II score for the study sample was 1.97 (95% CI: 1.75–2.19). The subscale scores were 2.27 (95% CI: 2.02–2.52) for “Physiological Effects,” 1.49 (95% CI: 1.25–1.74) for “Communication,” 1.83 (95% CI: 1.53–2.12) for “Harmful Effects,” 3.07 (95% CI: 2.61–3.54) for “Disease Progression,” and 1.21 (95% CI: 0.95–1.47) for “Fatalism” (Table 3).

**Table 3.** The Japanese Version of the Barriers Questionnaire II Total Score and Subscales

	Baseline (***n*** = 46)	
	Mean	95% CI
JBQ-II total score	1.97	1.75–2.19
Subscale		
Physiological effects	2.27	2.02–2.52
Communication	1.49	1.25–1.74
Harmful effects	1.83	1.53–2.12
Disease progression	3.07	2.61–3.54
Fatalism	1.21	0.95–1.47

CI, confidence interval; JBQ-II, Japanese version of the Barriers Questionnaire-II.

### Comparison of barriers before and after opioid induction

The mean JBQ-II scores at baseline and after (day seven) were 1.90 (95% CI: 1.77–2.03) and 1.85 (95% CI: 1.71–1.99), respectively (Table 4). There was a significant difference in the subscale of Disease Progression score (mean 2.97 vs. 2.59; difference in means 0.38; standard error 0.16, *p* = 0.026). The subscale of fatalism showed a 34% and 47% change in effect size and standardized response mean, respectively.

**Table 4.** Comparison of Psychological Barriers Before and After the Induction of Opioids (*n* = 36)

	Mean (95% CI)		Difference		SRM	ES	Paired ***t*** statistics	***p***
	Baseline	After	Mean	SE				
JBQ-II total score	1.90 (1.77–2.03)	1.85 (1.71–1.99)	0.05	0.09	0.12	0.02	0.498	0.621
Subscale								
Physiological effects	2.22 (2.04–2.40)	2.10 (1.92–2.28)	0.12	0.12	0.23	0.05	0.976	0.336
Communication	1.38 (1.16–1.61)	1.43 (1.20–1.66)	−0.04	0.16	−0.06	−0.03	−0.27	0.785
Harmful effects	1.81 (1.58–2.04)	1.75 (1.52–1.98)	0.06	0.16	0.08	0.03	0.35	0.731
Disease progression	2.97 (2.73–3.21)	2.59 (2.36–2.83)	0.38	0.16	0.55	0.13	2.33	0.026^***^
Fatalism	1.06 (0.79–1.32)	1.42 (1.16–1.68)	−0.36	0.18	−0.47	−0.34	−1.99	0.055

^***^ *p* ≤ 0.05.

SRM, standardized response mean; (Baseline − After)/SD (Baseline − After); ES: effect size; (Baseline − After)/SD (Baseline).

### PPG achievement and the relationships between the barriers

PPG was successfully achieved in 24 (52.2%, 95% CI: 38.1–65.9) eligible patients with cancer pain. Among the patients who did not achieve PPG, 22 (47.8%, 95% CI: 34.1–61.9) and 15 (32.6%, 95% CI: 20.9–47.0) completed and failed, respectively. The mean baseline JBQ-II score of patients who achieved PPG during the study period was 1.88 (95% CI: 1.56–2.21). The mean JBQ-II score for patients who completed and did not achieve PPG and for patients who discontinued and did not achieve PPG was 2.08 and 1.94, respectively (Table 5).

**Table 5.** Relationship Between Psychological Barriers to Opioids, Regret About Decision to Use, and Achievement of Pain-Relief Goals

			PPG			
	Achieved (***n*** = 24)		Not achieved (***n*** = 22)			
			Include discontinuation^a^ (***n*** = 22/22)		Exclude discontinuation^b^ (***n*** = 15/22)	
	**Mean**	**95% CI**	**Mean**	**95% CI**	**Mean**	**95% CI**
JBQ-II total score	1.88	1.56–2.21	2.08	1.76–2.39	1.94	1.55–2.33
Subscale						
Physiological effects	2.20	1.81–2.59	2.37	2.03–2.71	2.06	1.81–2.71
Communication	1.43	1.05–1.81	1.56	1.22–1.91	1.28	0.88–1.68
Harmful effects	1.58	1.17–1.98	2.11	1.67–2.54	2.03	1.45–2.62
Disease progression	3.18	2.50–3.87	2.95	2.27–3.64	2.91	2.06–3.76
Fatalism	1.10	0.70–1.49	1.29	0.93–1.65	1.20	0.75–1.65

^a^ Includes discontinuation: patients who did not achieve PPG and whose Brief Pain Inventory score could be confirmed until study completion.

^b^ Exclude discontinuation: Patients who did not achieve PPG and terminated during the study.

### Patient Global Impression Scale-Severity

The number of patients in the PGI-S 0–1 (no resistance) and 2–6 (with resistance) groups were 16 and 20 patients, respectively. Their DRS scores were 24.4 (95% CI: 16.5–32.3) and 31.7 (95% CI: 24.2–39.1), respectively. The PPG achievement rate in the two groups was 62.5% and 55.0%. When grouped with PGI-S score, the baseline JBQ-II total score was significantly higher in the 2–6 (with resistance) group (mean, 1.54 vs. 2.10; the difference in means, 0.6; 95% CI: 0.1–1.0; *p* = 0.025) (Table 6).

**Table 6.** Comparison of Respective Scores According to Patient Global Impression Scale-Severity

	PGI-S		Difference		*p*
	0–1 (***n*** = 16)	2–6 (***n*** = 20)	Mean	95% CI	
DRS total score, mean (95% CI)	24.4 (16.5 to 32.3)	31.7 (24.2 to 39.1)	7.3	−3.6 to 18.1	0.181
JBQ-II total score, mean (95% CI)	1.54 (1.18 to 1.91)	2.10 (1.78 to 2.43)	0.6	0.1 to 1.0	0.025^*^
Subscale, mean (95% CI)					
Physiological effects	1.76 (1.30 to 2.23)	2.37 (1.96 to 2.79)	0.6	0 to 1.2	0.054
Communication	1.11 (0.63 to 1.58)	1.68 (1.25 to 2.11)	0.6	−0.1 to 1.2	0.075
Harmful effects	1.43 (0.90 to 1.95)	2.01 (1.53 to 2.48)	0.6	−0.1 to 1.3	0.102
Disease progression	2.29 (1.62 to 2.96)	2.83 (2.23 to 3.43)	0.5	−0.4 to 1.4	0.231
Fatalism	1.25 (0.82 to 1.68)	1.55 (1.17 to 1.93)	0.3	−2.7 to 0.9	0.296
PPG achievement rate, *n* (%)	10 (62.5%)	11 (55.0%)	7.5%	−23.1 to 35.7	0.741

^*^ *p* ≤ 0.05.

## Discussion

BAROC is the first Japanese multicenter study to assess the relationship between psychological barriers to opioid analgesic use and cancer pain.

Patients enrolled in the study accounted for >60% of all gastrointestinal and lung cancer cases, and the data reflected the actual clinical practice in Japan.^29^ Another feature of the trial is that eligibility was not restricted by performance status. Even patients in financially inadequate conditions could participate in the study. The study enrolled 26% of patients with an ECOG PS ≥3. Ideally, patients with PS ≥3 are excluded from clinical trials. However, there are clinical cases in which activities are severely restricted even in the absence of impaired consciousness, such as in the case of spinal cord involvement in cancer. The inclusion of financially deficient patients using strong opioid analgesics indicates that our data reflect real-world data.

The total JBQ-II score at baseline was similar to the psychological barrier score in a three-country European study on patients who had used opioids for an average of five months.^12^ We initially predicted that psychological barriers during opioid induction would be greater than those during administration, but the total scores were similar in both the cases. However, the Fatalism subscale score in this study showed a higher trend than that in previous reports.

Furthermore, Fatalism tended to increase further after one week of induction. This increase tended to be higher for those who had not achieved PPG than for those who had. The Fatalism subscale score indicates the belief that pain must be endured. In patients who were introduced to strong opioid analgesics for cancer pain but did not achieve PPG, this may reflect resignation to inadequate efficacy, as the goal was not reached after initiation of strong opioid analgesics.

Furthermore, the Disease Progression subscale score exists only for JBQ-II. It showed a significant decrease before and after the introduction of opioid analgesics, with no change in the total score. Several Japanese surveys have reported that pain progression and analgesic use are anticipated in cancer progression.^7,13^

The validation study of the JBQ-II also found that the Disease Progression subscale score has been shown to be potentially influenced by the patient's psychological state, symptoms, and general condition.^18^ Patients are highly aware of the progression of their illness when their doctor recommends the introduction of opioids; however, the subsequent decrease in the Disease Progression subscale score may reflect the patient's acceptance of his or her disease status over time.

This suggests that psychological barriers to opioid analgesics are complex, and the quality of concerns may change over time. It is crucial to have a detailed understanding of the individual patient's condition to manage psychological barriers and provide appropriate interventions.

PPG achievement can be regarded as an appropriate indicator of well-managed pain relief. Approximately half of the participants achieved PPG during the observation period. Several studies have reported PPG achievement rates of ∼30–50%, similar to that observed in this study.^19,30^ It has also been suggested that with appropriate opioid dose adjustment, pain is controlled within seven days in most patients. Based on these studies, it is reasonable to assess PPG at seven days. Psychological barriers at baseline for patients who achieved PPG and those who did not differ by 0.5 points on the subscale of harmful effects.

This may be due to concerns about adverse events from opioid initiation and the lack of active use of painkillers, which may have prevented patients from achieving PPG. This may also be due to psychological factors such as anxiety about opioid use, affecting compliance and the degree of pain relief.^31^ Adverse events caused by opioids affect adherence to the prescribed dosing regimen and the level of pain relief.^32^ Therefore, the percentage of patients achieving PPG may increase if adequate explanation on dealing with adverse events and support systems is provided at the point of opioid initiation.

Decision regret due to the introduction of opioid analgesics was also measured. The mean DRS score after adjuvant treatment for breast cancer was 10.6, with most participants reporting no regrets. In contrast to adjuvant therapy, cancer pain treatment is symptomatic and insufficient time is allowed for decision making. It has been reported that being forced to make decisions without sufficient information and understanding of disease status and treatment options leads to patient dissatisfaction.^33^

Factors that have been reported to influence decision making include the content and manner of explanation provided by the physician,^34,35^ the decision-making situation,^36^ involvement in the decision-making process, and the patient's beliefs and attitudes^37^ regarding the illness and treatment. It has also been reported that patients with no treatment options experience significantly increased physical pain, lower mental health scores, and lower overall satisfaction.^38^ It is possible that shared planning of analgesic use between clinicians and patients in advance may reduce regrets associated with pain management.

This study has several limitations. First, this is an exploratory hypothesis-generating observational study. Initially, 200 patients were expected to be enrolled in the survey, but the number of enrolled patients was much lower than expected. This may be due to a decrease in the number of patients eligible for the administration of strong opioid analgesics (a reduction of 20% from the previous year) since the start of registration, primarily due to a decrease in the number of patients assessed due to the COVID-19 epidemic.

Countries have reported that the COVID-19 pandemic has significantly reduced opportunities for cancer screening, treatment, and surgery.^39,40^ As this is an exploratory observational study, it was decided not to base the enrolment numbers on statistical evidence^26,41^ but to revise the enrolment numbers based on the actual enrolment numbers at the time of the protocol revision. The target enrolment number was set to 50 cases, considering enrolment at other centers is becoming possible.

Owing to the small number of cases, it cannot be ruled out that there may not have been a significant difference in the JBQ-II total score before and after the introduction of the system. In addition, 15% of the participants dropped out of the study without achieving PPG. As such, it was difficult to conduct a detailed analysis of the relationship between PPG achievement and psychological barriers.

Second, as an observational study, we could not specify the explanations that should be given to patients before starting strong opioid analgesics or the venue for such explanations, which follows the protocols of the individual centers in this regard. Psychological barriers may fluctuate depending on how the explanation is provided and the environment in which it is provided. In some situations, treatment must be initiated despite significant barriers, as quality of life is significantly reduced without opioid analgesics, even if the pain becomes more intense.

This study was conducted in a population that was already undergoing treatment. Therefore, the results of this study cannot be applied to populations in which the use of strong opioid analgesics has not yet been explored. Third, patients with cognitive impairment or psychiatric disorders and those unable to operate a smartphone or tablet were excluded. Therefore, it was not possible to enroll all patients receiving strong opioid analgesics.

## Conclusion

This study showed that patients with advanced recurrent cancer have psychological barriers to opioid induction. The relationship between the presence of psychological barriers before and after the induction of opioid analgesics and the speed of pain improvement was determined. The results may provide essential information for prospective intervention studies to develop individualized education programs for patients with psychological barriers to opioids.

## Data Availability

The datasets used and/or analyzed during this study are available from the corresponding author on reasonable request.

## Abbreviations Used

BAROC: Psychological Barriers to the Use of Opioid Analgesics for Treating Pain in Patients with Advanced Recurrent Cancer: A Multicenter Cohort Study
BPI: Brief Pain Inventory
BPI-SF: Brief Pain Inventory-Short Form
BQ: Barriers Questionnaire
BYOD: Bring Your Own Device
CI: confidence interval
COVID-19: Coronavirus Disease 2019
DRS: Decision Regret Scale
ECOG: Eastern Cooperative Oncology Group
ePRO: electronic patient-reported outcomes
JBQ-II: Japanese version of the Barriers Questionnaire II
NRS: Numerical Rating Scale
PGI-S: Patient Global Impression Scale-Severity
PMI: Pain Management Index
PPG: Personalized Pain Goal
PRO: Patient-Reported Outcome
PS: Performance Status
SAS: Statistical Analysis System
SD: standard deviation
